# Influence of Cu Content Variation on the Tribological Properties of Ni60CuMo with Sandwich-Structured Composite Coatings by Laser Cladding

**DOI:** 10.3390/mi15121429

**Published:** 2024-11-27

**Authors:** Fengqin Ji, Xincheng Li, Songyang Zhang, Ming Pang

**Affiliations:** College of Aeronautical Engineering, Civil Aviation University of China, Tianjin 300300, China

**Keywords:** additive manufacturing, Ni60CuMo, self-lubrication, wear resistance, RuT400

## Abstract

To enhance the tribological properties of the coatings and to inhibit cracking, sandwich-structured composite coatings were fabricated, consisting of a Ni60CuMo/IN718 transition layer and a Ni60CuMo/Ni-coated Cu wear-resistant layer with four different Ni-coated Cu contents. The results indicate that the transition layer inhibits the crack formation in the coating, and the refined grain structure stabilizes its average hardness at approximately 485 HV_0.5_. Increasing the Cu content in the wear-resistant layer exacerbates the segregation of the Cu-rich solid solution phases and refines the in situ-generated Cr_7_C_3_, TiC, and NbC phases. The average hardness of the wear-resistant layer decreases from 474 HV_0.5_ to 408 HV_0.5_ as the Ni-coated Cu content increases from zero to 75%. The coating with 50% Ni-coated Cu has the best Cu self-lubricating properties and exhibits the best wear resistance at both room and high temperatures. At room temperature, abrasive wear is the primary wear mechanism in the coatings. Although the ductility of the coatings is improved with increasing Cu content, excessive Cu reduces the hardness and load-bearing capacity. At 300 °C, oxidation wear becomes the dominant wear mechanism, accompanied by plastic deformation and three-body wear as the Cu content increases. At 500 °C, severe oxidation wear is the dominant mechanism, with excessive Cu leading to oxidation film failure.

## 1. Introduction

Due to its excellent castability, damping capacity, and machinability, cast iron is widely used to manufacture various parts and machinery, including critical components such as valve seat rings [1]. As a vital part of the engine’s valvetrain, the valve seat ring plays a key role in sealing and heat transfer [2]. However, cast iron valve seat rings are prone to failure or damage due to severe surface wear when exposed to high temperatures, high pressure gas impacts, and the friction caused by high speed, frequent valve striking [3,4]. Therefore, surface protection treatments are required for cast iron valve seat rings [5]. Laser cladding technology uses a high energy density laser beam to rapidly melt and cool the coating and substrate to produce a coating that meets practical application requirements. This method offers advantages such as a small heat-affected zone, high bonding strength, and precise controllability [6,7].

However, the high carbon content and poor weldability of cast iron present significant challenges to the production of wear-resistant coatings by laser cladding. The high carbon content in the cast iron substrate can lead to carbon migration during laser cladding, resulting in the formation of a large amount of carbide precipitates in the coating area [8]. Sommer et al. [9] found that the rapid cooling and high temperature gradients associated with laser cladding induce residual stresses in the coating. The brittle microstructure within the coating can become crack initiation sites under the influence of these residual stresses. Nickel-based alloy powders are considered one of the preferred materials for cladding iron-based coatings due to their high hardness, excellent wear resistance, self-fluxing properties, and good weldability on various substrates [10]. Li et al. [11] observed that laser remelting can eliminate defects such as cracks and pores in nickel-based coatings on gray cast iron surfaces. Yi et al. [12] found that reducing the laser scanning speed during cladding on cast iron substrates refines carbide grains, suppresses the uneven distribution of graphite phases, and reduces crack formation. In previous research, a Ni60CuMo/IN718 nickel-based composite cladding coating was applied to the surface of vermicular cast iron, and crack-free coating was achieved by controlling the laser power [13]. However, the excessive migration of the substrate elements led to the significant dilution of the cladding material. While the wear resistance of a coating is maintained by the oxidation of Fe and Ni and the in situ formation of hard carbide phases, there are limitations in terms of self-lubrication performance.

Various self-lubricating materials are currently available for the self-lubrication of coatings, including traditional organic lubricants and solid lubricants such as transition metal dichalcogenides(TMDs), h-BN, and soft metals such as Cu and Ag. However, organic lubricants are unsuitable for high temperature environments and may be limited by specific environmental requirements for lubricants [14]. Organic lubricants are generally unsuitable for high-temperature applications, such as in aerospace engines and high-temperature metallurgical equipment, because organic lubricant phases tend to decompose, evaporate, or carbonize under high temperatures, leading to lubrication failure. In oxidizing environments, organic lubricants are prone to oxidation, and in oxygen-rich air, their molecular structures may be disrupted, producing by-products with no lubricating properties. Under high pressure or shear conditions, organic lubricants may be unable to maintain an intact lubricating film, making them susceptible to extrusion or rupture, which prevents sustained lubrication and adversely affects their lubricating performance [15,16]. Additionally, solid lubricants such as sulfides and fluorides are prone to thermal decomposition at the high temperatures of the cladding process, reducing their effectiveness [17]. Liu et al. [18] found that h-BN, as a high temperature solid lubricant, performs better under elevated temperature conditions. Moreover, the high cost of soft metal lubricants like Ag poses a challenge to the overall cost of cast iron coatings. Therefore, Cu is widely used in the manufacture of self-lubricating coatings due to its high thermal conductivity, ductility, and relatively low cost. Zheng et al. [19] significantly improved the wear resistance of coatings by adding Cu-coated MoS_2_ to Ni60. Yang et al. [20] varied the Cu content in the high-entropy alloy FeCoCrNiCu_x_ and found that increasing the Cu content gradually reduced the friction coefficient and improved the strength and wear resistance of the coating. Zhu et al. [21] added Cu to Co-based coatings and achieved better friction reduction and wear resistance at room temperature. However, when the Cu content in the coating is too low, the lubrication properties of the coating are limited. When the Cu content is low, the dilution rate of the cast iron substrate decreases, allowing more iron from the substrate to be incorporated into the coating. This prevents Cu from forming a sufficiently thick or continuous lubricating film, making it difficult to fully cover the friction surface and resulting in a discontinuous lubrication layer, which weakens the lubrication effect on the friction surface. In many corrosive environments, the dilution of iron in the coating reduces the corrosion resistance of the coating [22]. Rasch et al. [23] found that the application of Ni-Cu coatings in welding ductile iron is limited, as low Cu content combined with elevated interface temperatures increases the likelihood of carbon atoms from the cast iron substrate diffusing into the coating. This can lead to carbide formation within the coating, making it prone to thermal cracking in high-carbon conditions. On the contrary, when the content is too high, it will inevitably bring about the sacrifice of the coating hardness, and then affect the coating performance.

In the manufacture of wear-resistant coatings with added Cu, it remains challenging to effectively address the issue of the migration of cast iron substrate elements, and the dilution of the lubricating phases in the coating is an unavoidable difficulty. Therefore, it is necessary to optimize the structure of the composite coating by introducing a transition layer to mitigate these problems. Lu et al. [24] alleviated the differences in the thermal properties between a titanium alloy substrate and a Ni-based coating by incorporating a V-Cr transition layer. The V element was used to mitigate the differences between the two layers, while the Cr element acted as a bridge, dissolving infinitely with V on the one hand and achieving solid solution with most elements in the Ni-based alloy coating on the other. Xue et al. [25] solved the bonding problems between an iron-based H13 steel and a Fe104 alloy by using a Ni25 transition layer. The diffusion of Ni from the Ni25 transition layer improved the toughness of the Fe104 coating and helped to reduce carbide formation within the coating. Li et al. [26,27] introduced a NiCu alloy as a transition layer in the coatings, which improved the bonding performance between the Ni-based coating and the cast iron substrate, and suppressed crack formation at the interface. According to the previous research [28], crack-free Ni60CuMo/IN718 with certain wear-resistant properties as a transition layer can inhibit the migration of substrate elements. The Ni, Cr, Nb, and other metallic elements in the transition layer consumed the elements Fe and C diffused from the substrate, inhibiting the diffusion of partial substrate elements. And this prevented the uneven distribution of hard carbides into the coating due to the upward diffusion of the carbon elements from the substrate, reducing the risk of cracking and significant hardness fluctuations. Also, the bonding properties between the Cu-containing nickel-based coating and the cast iron substrate can be improved using this method [13].

To solve the problem of coating cracking on cast iron substrates and to optimize the lubricating and wear-resistant properties of these coatings, sandwich-structured Ni60CuMo/Ni-coated Cu composite coatings with a crack-free Ni60CuMo/IN718 transition layer were fabricated on the surface of RuT400 by laser cladding. The influence of Cu content on the lubricating and wear-resistant properties of the composite coatings and the related wear mechanism were investigated by subtracting the ratio of Ni-coated Cu from the Ni60CuMo in the wear-resistant layer. This study can provide data support for engineering parameter optimization and new insights into coating structure improvements.

## 2. Experimental Procedures

### 2.1. Materials and Methods

The substrate material selected for this study is vermicular cast iron (RuT400), which was chosen to match practical application contexts. The basic dimensions of the substrate samples were 75 mm × 55 mm × 10 mm. The chemical composition of RuT400 is listed in Table 1. The primary cladding material chosen was Ni60CuMo alloy powder (China, Chengdu, Huayin) with a particle size range of 150–300 mesh. Additionally, Inconel 718 (IN718) powder and Ni-coated Cu powder (China, Beijing, HuaWeiRuiKe) with a particle size range of 150–300 mesh, were also added to the transition layer and the wear-resistant layer, respectively. The chemical compositions of these three powders are listed in Table 2.

**Figure 1:**
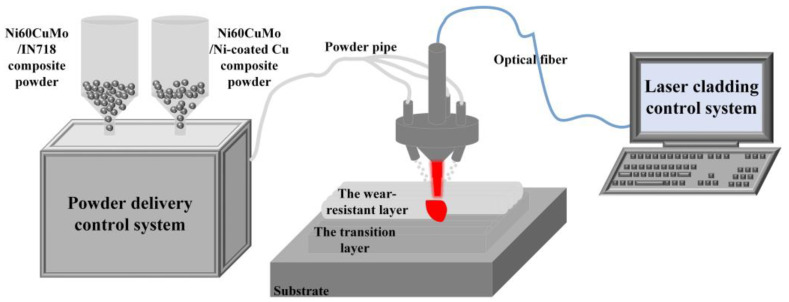
Schematic of laser cladding of multilayer structural coatings.

Previous research has demonstrated that the Ni60CuMo/IN718 composite coating effectively suppresses cracking and exhibits the best wear resistance at a laser power of 1400 W. Therefore, the coating fabrication in this study is based on the established laser power. Additionally, the coating structure was optimized to improve the lubricating and wear-resistant properties. This optimization included the selection of Ni60CuMo/IN718 as the transition layer and the use of Ni60CuMo/Cu as the wear-resistant layer. To avoid the high reflectivity of pure Cu, and the low density of the Cu lubricant phase and its tendency to float and unevenly distribution during the cladding process, Ni-coated-Cu was used to vary the Cu content of the wear-resistant layer. The compositions of the powders are detailed in Table 3, with the lap rate of 65%, the scanning speed of 7 mm/s, and the spot diameter of 4.2 mm. To prevent oxidation during the laser cladding process, high purity Ar was used as the protective gas. The coatings were prepared using an RFL-3000S fiber laser from Wuhan Rike Co., Wuhan, China. Figure 1 shows a fabrication schematic for the Ni60CuMo/IN718 transition layer and the Ni60CuMo/Ni-coated Cu wear-resistant layer. For convenience, the coatings produced with four different Cu contents are abbreviated as C1, C2, C3, and C4 coatings, respectively.

### 2.2. Material Characterization

Penetrant testing is commonly used to detect cracks within coatings. After penetrant testing, any red regions that appear on the white surface indicate the presence and distribution of cracks within the coating. The coated specimens were cut into metallographic samples with dimensions of 10 mm × 5 mm × 8 mm perpendicular to the laser scanning direction using a wire-cutting machine (Taizhou Zhongxin, Taizhou, China). These samples were then mounted in a metallographic mounting machine (XQ-1) at 135 °C. Subsequently, the mounted samples were ground and polished on an automatic grinding and polishing machine (ZMP-1000, Zhongke, Tianjin, China) using 60–2000 mesh sandpaper and diamond polishing paste. The polished samples were then etched using a prepared etching solution (HCl:HNO_3_:HF = 1:3:3) for 30–40 s, followed by rinsing with alcohol and drying. The etched samples were observed for microstructural and elemental analysis using a Phenom XL scanning electron microscope (SEM) (Hitachi, Tokyo, Japan) equipped with energy dispersive spectroscopy (EDS).

Phase detection experiments were usually performed using an X-ray diffractometer (XRD) (Rigaku D/max-2500/PC) (Tokyo Science, Tokyo, Japan) with the following basic parameters: Cu K-α radiation (λ = 1.54 Å), operating at a voltage of 40 kV and a current of 200 mA. The scanning range was set from 10° to 110° (2θ) with a scanning speed of 8°/min. Before the test, the XRD specimens with dimensions of 10 mm × 10 mm × 4 mm were cut from the coatings and then ground and polished with sandpaper and polishing liquid to minimize detection errors.

### 2.3. Mechanical Property Tests

Hardness measurements were carried out using an HVS-1000 Z microhardness tester (Lidun, Shanghai, China) under a load of 500 g and a dwell time of 10 s. Hardness measurements were taken every 50 μm from the surface of the coating down to the substrate. Before measuring the hardness distribution of the coating, the metallographic samples were reground and repolished.

The wear tests were conducted using an MPX-3G high-temperature friction and wear-testing machine (Hengxu, Jinan, China) under the following conditions: rotating speed of 300 r/min, rotating radius of 1.5 mm, test load of 30 N, and test duration of 40 min. Specimens with dimensions of 15 mm × 15 mm × 8 mm were prepared and polished with 60–2000 mesh sandpapers to ensure a smooth surface. The wear and lubrication performance of the coating were studied at room temperature (25 °C), 300 °C, and 500 °C. Si_3_N_4_ ceramic balls with a hardness of 1000 HV_0.5_ and a diameter of 6.35 mm were used as the grinding material. The coefficient of friction (COF) was automatically recorded by the instrument’s built-in data acquisition system. The wear volume of the coating was measured using a DC-700 surface profilometer (Dicheng Lianshuo, Tianjin, China). Subsequently, the worn surface morphologies and phase composition were characterized using SEM and EDS, respectively.

## 3. Results and Discussion

### 3.1. Macroscopic Morphology and Phase Analysis

**Figure 2:**
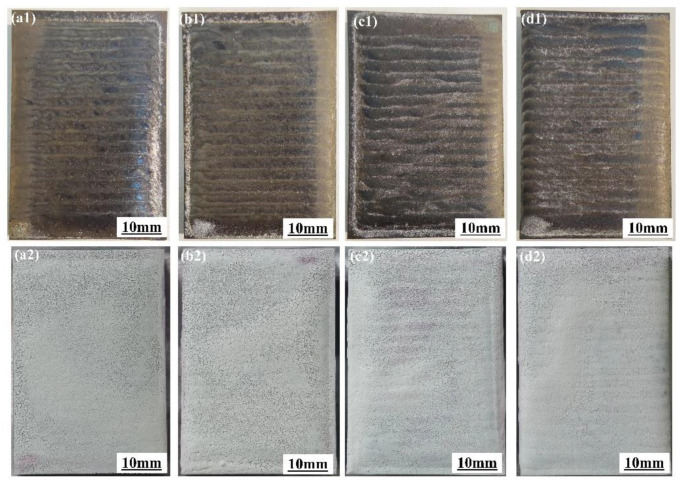
Macroscopic morphologies and penetration test results of the studied coatings for (**a1**,**a2**) C1 coating, (**b1**,**b2**) C2 coating, (**c1**,**c2**) C3 coating, and (**d1**,**d2**) C4 coating.

Figure 2(a1–d1) shows the surface morphology of four different sandwich-structured coatings with varying Cu contents, while Figure 2(a2–d2) shows the penetration test results. It can be observed from Figure 2(a1–d1) that the surfaces of all four cladding layers exhibit good qualities with no visible cracks or other defects. The inspection results presented in Figure 2(a2–d2) also confirm the absence of porosity and cracks in the coatings. The cladding material system of the wear-resistant layer is similar to that of the transition layer, which has an excellent cushioning effect, and the transition layer effectively suppresses the generation of cracks in the coating, and the similarity of the material system also reduces the susceptibility to cracking in the wear-resistant layer.

**Figure 3:**
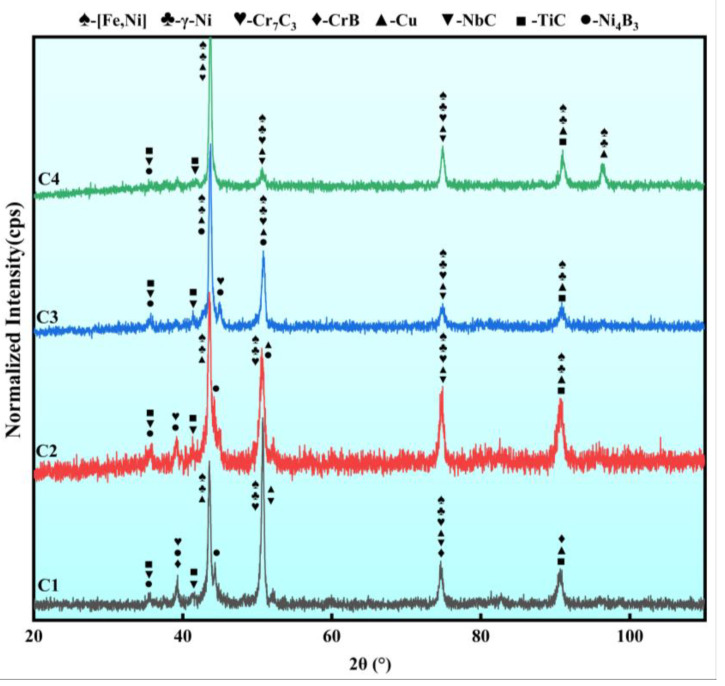
Normalized X-ray diffraction diagram of the four studied coatings with different Cu contents.

XRD was used to analyze wear-resistant composite coatings with different Cu contents. Since the XRD data showed relatively low peak intensities for carbides and borides, the obtained XRD data were normalized to enable accurate phase identification; the results from phase identification are shown in Figure 3. As shown in Figure 3, different Ni-coated Cu content in the wear-resistant layer does not significantly affect the phase composition of the coatings. However, the main diffraction peak at the 2θ = 43° position exhibited an increasing trend with the increase in Ni-coated Cu content. Notably, a new Cu diffraction peak (2θ = 96°) appeared in the C4 coating. This indicates that the lubricating phase Cu had not participated in the transient reactions within the molten pool to form other compounds [29]. The increase in Ni-coated Cu content effectively raised the amount of lubricating-phase Cu in the coating, resulting in the higher peak intensity of the diffraction peaks. Moreover, Figure 3 shows a shift in the main peak to the right with increasing Cu content. This shift indirectly reflects the decrease in the lattice size within the coatings. On the one hand, laser radiation generates thermal stress, which can cause lattice distortion of the crystals in the Ni60CuMo/Ni-coated Cu wear-resistant layer. On the other hand, lattice distortion or dislocations occur when Cu atoms occupy the lattice sites of other atoms in a solid solution, leading to a shift in the characteristic peaks [21]. The primary phases within the coatings include γ-Ni, [Fe,Ni] solid solution, and Cu dissolved in the matrix phase. Additionally, the coatings contained carbides such as Cr_7_C3, NbC, and TiC, as well as borides consisting mainly of Ni_4_B_3_ and CrB. These in situ-formed hard phases not only increased the hardness of the coatings, but also ensured strong bonding with the coating matrix. This prevented the stress concentration that could arise from excessively high local hardness [25].

### 3.2. Microstructure Characterization

**Figure 4:**
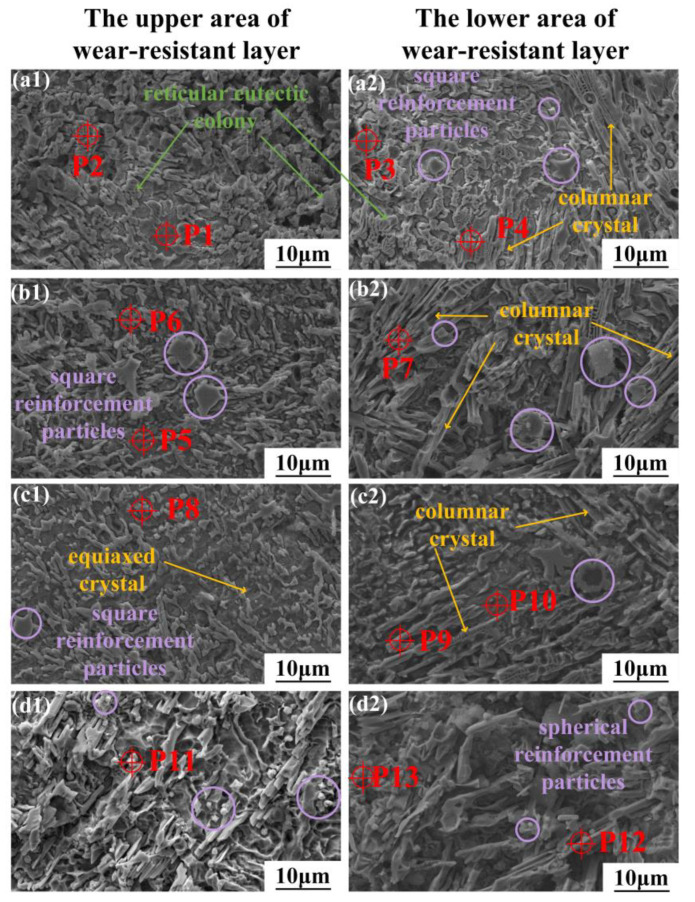
The microstructure of the four wear-resistant layers with different Cu contents. (**a1**–**d1**) is the upper area for C1–C4 coatings, (**a2**–**d2**) is the lower area for C1–C4 coatings.

Figure 4 illustrates the microstructural characteristics at the upper and lower regions of the wear-resistant layer within the coating. The EDS analysis results for the characteristic points within wear-resistant layers for the four different Cu contents are listed in Table 4. In the wear-resistant layer without added Ni-coated Cu (Figure 4(a1,a2)), an amount of reticular eutectic clusters (P2) was observed, which was identified as the Cr_7_C_3−_dominated eutectic structure [30]. The eutectic structure formed during the early solidification phase when the hard phase had not yet fully precipitated. Additionally, high concentrations of Cr_7_C_3_ (P4) were detected on the columnar dendrites in the lower region, whereas the Cr content in the matrix phase of the coatings was substantially reduced, with the formation of mainly [Fe, Ni] (P1) solid solutions.

The Cu element tends to remain in coatings as a solid solution [31]. The increase in Cu content in the wear-resistant layer is beneficial to the toughness and lubrication properties of the coating, and improves its crack resistance and wear performance. Moreover, many square and spherical particles were observed in these wear-resistant layers, and the EDS data results indicated the significant enrichment of the Ti, Nb, and C elements within these particles, which were considered to be the TiC- and NbC-reinforced phase eutectics, with the results shown in the XRD of Figure 3. TiC and NbC phases have the same face-centered cubic structure and similar lattice constants (TiC: 0.424 nm, NbC: 0.447 nm). Both carbides also exhibit significantly higher melting points (TiC: 3160 °C, NbC: 2300 °C) in the molten pool than in other phases [32,33]. The molten pool temperature and the cooling rate influence the growth morphology of [TiC, NbC] particles [34].

The microstructure of the wear-resistant layer exhibited a trend of gradual refinement with the increase in Cu content. In the lower region of Figure 4, the dendritic structures clearly show that the grain size became progressively finer and sparser, eventually forming fine needle-like chromium carbides (Figure 4(d2)). Additionally, the morphology of [TiC, NbC] in the wear-resistant layer changed from small square particles in C1 to small spherical hard phase particles in C4, further illustrating the trend of grain size refinement within the coatings. As shown in Figure 4, the microstructural dimensions of the lower area were generally larger than those of the upper area. The lower area contained more and larger columnar grains, while the upper area had a more compact and finer microstructure. This phenomenon of grain refinement from the bottom to the top of the coating is known as the “skin effect” during the laser cladding process [35]. According to the solidification kinetics [36], the shape control factor K and the liquid phase composition are the main factors affecting crystal morphology, where the shape control factor K is determined by the ratio G/R, and G is the temperature gradient, and R is the solidification rate. As the K value decreases, the grains gradually transition from planar to dendritic and eventually to equiaxed crystals. In the lower region near the interface between the coating and substrate, the higher G value promotes the growth of the dendritic structures along the heat dissipation direction. Conversely, in the upper region of the coating, the solidification rate is higher due to exposure to air, resulting in a shorter crystal growth time and the formation of smaller equiaxed grains [37].

**Figure 5:**
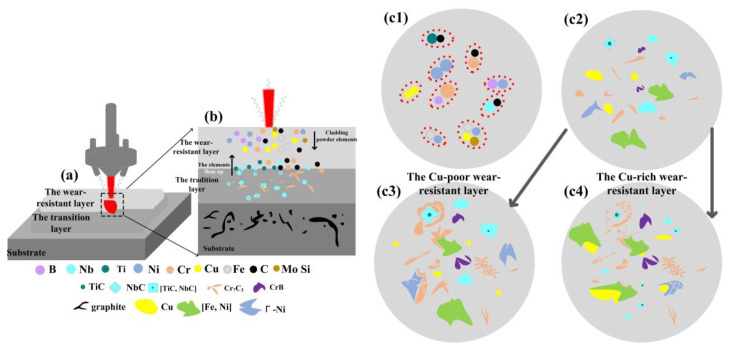
The mechanism of microstructure forming during the preparation of the wear-resistant layer. (**a**) the laser cladding processes of transition layer and wear-resistant layer, (**b**) the Ti and Nb elements in the transition layer diffused into the wear-resistant layer, (**c1,c2**) the elements floating up from the transition layer form the reinforced phases in the molten pool of the wear-resistant layer, (**c3**) minimal Cu segregation in the Cu-poor wear-resistant layer, (**c4**) pronounced Cu segregation in the Cu-rich wear-resistant layer.

Figure 5 illustrates the mechanism of microstructure formation within the molten pool and coating during the fabrication of the wear-resistant layer. The wear-resistant layer was fabricated on the base formed by the previously prepared transition layer (Figure 5a). According to the data in Table 2, the Ti and Nb elements were only present in the transition layer powder IN718, while the [TiC, NbC]-reinforced phases were also detected, as shown at P3 and P 11 in Figure 4. This indicates that the elemental floating up phenomenon occurred in the transition layer during the fabrication of the wear-resistant layer. The TiC and NbC phases formed in the transition layer decomposed and diffused into the molten pool of the wear-resistant layer, thus introducing elements such as Ti and Nb into the wear-resistant layer. These elements then led to the formation of TiC- and NbC-reinforced phases in the wear-resistant layer (Figure 5b). As seen in Figure 5(c1,c2), the elements floating up from the transition layer and the cladding powder reacted transiently in the molten pool and formed the reinforced phases, such as TiC, NbC, Cr7C3, CrB together with γ-Ni, [Fe, Ni] solid solution, and Cu in the solid solution state. However, the microstructures of the wear-resistant layers with the different Cu contents were affected by Cu segregation, resulting in different morphologies. In Figure 5(c3), the Cu-poor wear-resistant layer shows minimal Cu segregation in the solid solution phase, allowing sufficient time for the phases formed in Figure 5(c2) to grow into larger grains. Conversely, in the Cu-rich wear-resistant layer shown in Figure 5(c4), the higher Cu content led to more pronounced Cu segregation and a more complex temperature field. The superior thermal conductivity of the Cu element caused more significant temperature variations in the regions near the segregation, which led to faster solidification and precipitation, and the formation of smaller grains in the surrounding regions. This resulted in the development of the formation of spherical [TiC, NbC] and needle-like Cr7C3-reinforced phases due to elemental floating up as well as the smaller equiaxed grains, shown in Figure 4c,d.

**Figure 6:**
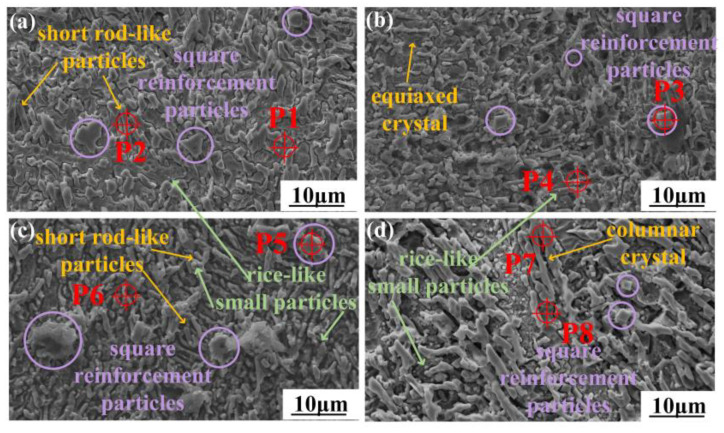
Microstructure of transition layers of the four studied coatings. (**a**) C1 coating, (**b**) C2 coating, (**c**) C3 coating, and (**d**) C4 coating.

Figure 6 depicts the microstructural characteristics in the transition layer region of the four studied composite coatings, and the EDS results of certain characteristic structures are listed in Table 5. Comparing the grain size in the wear-resistant layer shown in Figure 4, it is obvious that the microstructures within the transition layer of each coating exhibited a denser and finer morphology. In all four transition layers of the studied coatings, small square particles (such as P3 and P5) were present, as listed in Table 5, which were [TiC, NbC]-reinforced phases also found in the wear-resistant layer. As the Ti and Nb elements originated mainly from the transition layer-cladding powder IN718, this further confirms that the transition layer was remelted during the fabrication of the wear-resistant layer, which resulted in the elemental diffusion between the transition layer and the molten pool of the wear-resistant layer. Through this diffusion, NbC could be detected even in the upper region of the wear-resistant layer, an observation that indirectly confirms the good metallurgical bonding between the transition layer and the wear-resistant layer. Additionally, dispersed short rod-like particles (P2, P6), small dendrites (P7), and rice-like small particles (P4) were observed in the transition layer, shown in Figure 6. EDS analysis results indicate that the short rod-like phases consisted mainly of Cr and C elements, while the small rice-like particles contained large amounts of C and other metal elements. The temperature of the transition layer rapidly increased again due to the reheating effect caused by the subsequent layers, producing a tempering-like effect [38]. This tempering effect, induced by accumulated heat, caused the solute phases originally present in the solid solution of the transition layer and later remelted to exhibit carburization characteristics during solidification. As a result, rice-like carbide particles precipitated in the transition layer.

### 3.3. Hardness Analysis

**Figure 7:**
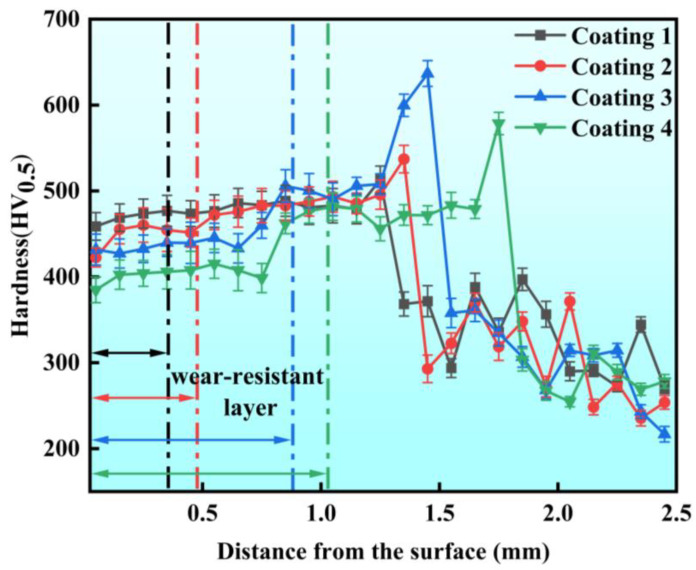
Hardness distribution at different locations from the surface of the coating to the substrate.

Figure 7 shows the hardness distribution from the coating surface to the substrate at different positions. The average hardness values of the four wear-resistant layers with different Cu contents (C1–C4) were 474 ± 12 HV_0.5_, 459 ± 18 HV_0.5_, 439 ± 15 HV_0.5,_ and 408 ± 16 HV_0.5_, respectively. Compared to the average hardness of the substrate (244 ± 10 HV_0.5_), the hardness increased by 94.3%, 88.1%, 79.9%, and 67.2%, respectively. Additionally, the hardness in the transition layer remained relatively stable at approximately 485 ± 8 HV_0.5_, with a sharp increase in the heat-affected zone (HAZ), before decreasing rapidly in the substrate region. As the Cu content increased, the hardness of the wear-resistant layer decreased. For the C1 coating without the Ni-coated Cu, the average hardness of the wear-resistant layer was close to that of the transition layer. However, the difference in hardness between the wear-resistant layer and the transition layer became more pronounced with the increasing Cu content in the C2–C4 coatings. According to the mixture rules of composite materials, the addition of soft phases like Cu to the hard materials decreased the overall hardness of the coating [39]. As the Cu content increased, severe Cu segregation occurred within the coating. The relatively large atomic radius of the Cu atoms weakened the lattice distortion within the solid solution and reduced the strengthening effect of the solid solution, thus decreasing the hardness of the coatings [40].

In the interface region between the wear-resistant layer and the transition layer, a gradual increase in hardness could be observed, shown in Figure 7. This is attributed to the re-diffusion of the elements during the secondary remelting of the transition layer. The pre-formed carbides in the transition layer were melted during the laser cladding process of the wear-resistant layer and participated in the molten pool reactions. These carbides provided additional carbon atoms to the wear-resistant layer. The diffusion of the elements led to more carbide phases precipitating at the bottom of the wear-resistant layer, resulting in the formation of more dendritic hard phases in the lower region of the wear-resistant layer and a steady increase in the hardness in the interface region, as shown in Figure 7. In addition, the transition layers also produced significant grain refinement after secondary laser remelting, shown in Figure 6, and this refined microstructure played a crucial role in increasing the hardness of the transition layer. During hardness testing, these fine grains effectively impeded dislocation motion and grain boundary sliding when pressure was applied by the indenter, resulting in higher hardness values [41]. Furthermore, according to the EDS analysis results of the transition layer listed in Table 5, the matrix phase has a relatively low Cu content, which effectively suppressed the hardness reduction typically caused by the softer Cu phase. As a result of these combined factors, the hardness of the transition layer was higher than that of the wear-resistant layer. Due to the phase transformation of the substrate’s iron-phase microstructure, which formed ledeburite and martensite containing retained austenite [42], the hardness significantly increased in the HAZ. The hardness progressively increased from the wear-resistant layer to the transition layer and then to the HAZ. This gradient ensured the wear resistance and lubrication effectiveness of the high-lubricity wear-resistant layer, the impact resistance of the high-hardness transition layer at the bottom of the coating, and the metallurgical bonding between the coating and the substrate.

### 3.4. Tribological Properties

**Figure 8:**
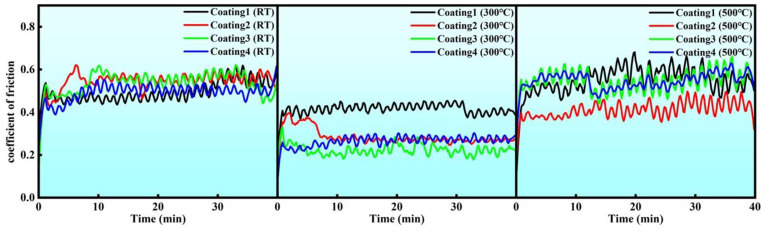
Coefficient of the friction curves of the studied coatings at RT, 300 °C, and 500 °C, respectively.

The coefficient of the friction (COF) curves of the four coatings with different Cu contents at room temperature (RT), 300 °C, and 500 °C are shown in Figure 8. The COF for each coating was measured on three samples and then averaged. During the initial wear stage, the coating surface and the corresponding counterface exhibited microscale asperity contact, which was characterized by a small contact area and high contact pressure. This led to extensive shearing of microprotrusions and the formation of wear debris. Typically, materials with lower strength and toughness require a longer running-in period during the initial wear phase to transition into a stable wear stage [43]. After entering the stable wear stage, the average COFs of the coatings C1 to C4 were 0.494 ± 0.032, 0.543 ± 0.021, 0.535 ± 0.026, and 0.492 ± 0.019 at RT, respectively, while the average COFs were 0.414 ± 0.017, 0.284 ± 0.011, 0.223 ± 0.009, and 0.264 ± 0.014 at 300 °C, respectively, and 0.548 ± 0.029, 0.410 ± 0.026, 0.545 ± 0.021, and 0.545 ± 0.018 at 500 °C, respectively. The larger fluctuations in the COF curves indicate that the coating surface was more prone to damage and plastic deformation due to the applied load [36]. At RT, the C1 coating without Ni-coated Cu exhibited higher hardness and better resistance to deformation from the counterface, and thus a more stable COF curve. However, at high temperatures, the hardness of the coatings with higher Cu content was significantly affected as the temperature increases. At 500 °C, the coatings tended to soften and were more prone to plastic deformation. The accumulation of frictional heat and the formation of oxide films at high temperature led to the periodic localized fracture, accumulation, and removal of these films, which caused severe fluctuations in the COF curve [44]. In contrast, at 300 °C, there was no significant softening of the coatings, and the friction layer retained the ability to resist the compressive and shear forces from the counterface. As a result, the fluctuation of the COF curves was more stable than at 500 °C. Additionally, the oxides produced by the oxidation of Ni and Cu at this temperature had a lubricating effect, altering the stress state of the friction surface and improving the tribological properties [43]. Consequently, the average COFs of the coatings at 300 °C were lower. Moreover, at 300 °C, the average COF initially decreased and then increased with the addition of Cu content, which indicates that excessive Cu content in coating can weaken its beneficial effect, leading to a negative feedback response [45].

**Figure 9:**
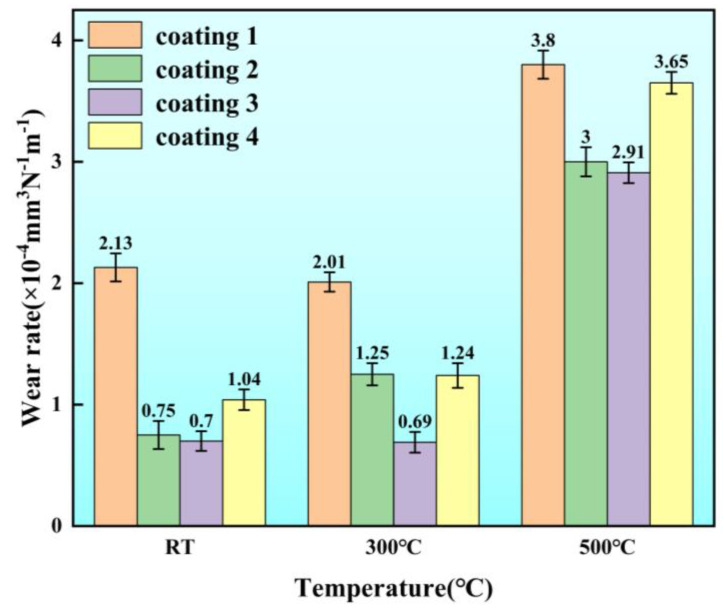
Wear rate of the studied coatings at room temperature, 300 °C, and 500 °C.

Figure 9 shows the wear rates of each coating at room temperature, 300 °C, and 500 °C. It can be observed that the wear rate initially decreased and then increased with the increase in Cu content. The C3 coating exhibited the lowest wear rate of 0.70 × 10^−4^ mm^3^N^−1^m^−1^, 0.69 × 10^−4^ mm^3^N^−1^m^−1^, and 2.91 × 10^−4^ mm^3^N^−1^m^−1^ at both RT and high-temperature conditions, respectively. The addition of Cu enhanced the tribological properties of the coatings and improved its lubricating properties, thus reducing the wear rate. However, the excessive addition of Cu decreased the load-bearing capacity of the coating and increased its plasticity, making the coating more susceptible to severe plastic deformation during wear, leading to more pronounced cutting actions and an ultimately higher wear rate [46]. Moreover, at 500 °C, the wear rate of the coatings increased significantly for all the coatings. This was likely due to the thermal softening of the coatings at 500 °C, which made them more prone to plastic deformation. The plastic deformation of the coatings led to grain compression, increasing the dislocation density, and causing twin boundary cracking, which promoted the delamination and spalling of the coating matrix and oxide products. Due to the inherently low SFE value of the Cu (SFE_Cu_ = 45 mJ/m^2^, SFE_Ni_ = 125 mJ/m^2^, SFE_Fe_ = 180 mJ/m^2^), the coatings with a high Cu content had lower overall SFE values. The lower SFE made the coatings more susceptible to twin boundary cracking [47]. This further explains the higher wear rate of C4 coating with the higher amount of Cu content.

### 3.5. Wear Mechanisms

**Figure 10:**
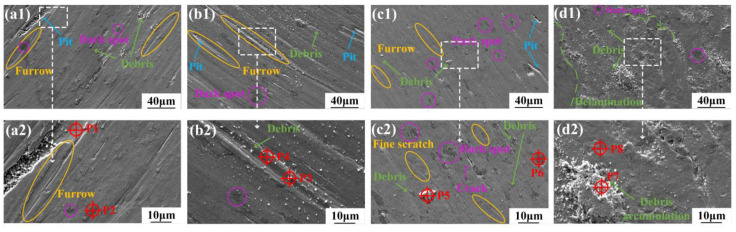
Worn morphologies of the studied coatings at room temperature. (**a1**,**a2**) C1 Coating, (**b1**,**b2**) C2 Coating, (**c1**,**c2**) C3 Coating, (**d1**,**d2**) C4 Coating.

**Figure 11:**
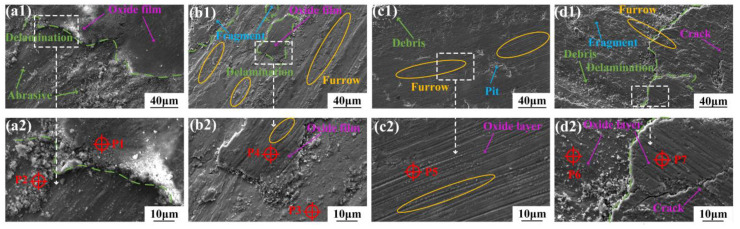
Worn morphologies of the studied coatings at 300 °C. (**a1**,**a2**) C1 Coating, (**b1**,**b2**) C2 Coating, (**c1**,**c2**) C3 Coating, (**d1**,**d2**) C4 Coating.

**Figure 12:**
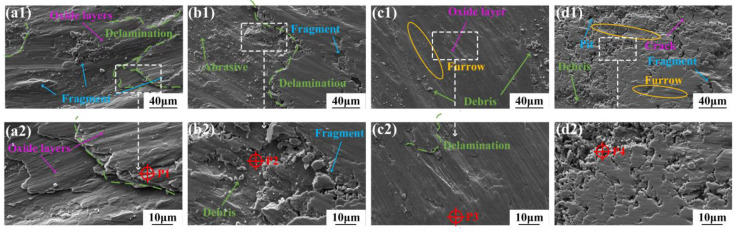
Worn morphologies of the studied coatings at 500 °C. (**a1**,**a2**) C1 Coating, (**b1**,**b2**) C2 Coating, (**c1**,**c2**) C3 Coating, (**d1**,**d2**) C4 Coating.

To understand the wear characteristics and intrinsic wear mechanisms of each coating under different conditions, Figure 10, Figure 11 and Figure 12 show the worn morphologies at RT, 300 °C, and 500 °C, where a2–d2 is the enlarged part of the white box in a1–d1 and Table 6, Table 7 and Table 8 list the related EDS analysis results, respectively. In Figure 10a, there are prominent furrows, wear debris, and spalling pits on the worn surface of the C1 coating, indicating clear abrasive wear. For the C2 coating, the furrows became denser and more pronounced, while the spalling pits decreased, but still showed typical abrasive wear characteristics (Figure 10b). The dark areas on the C3 coating (Figure 10c) became more prevalent as the Cu content increased. An EDS analysis of these areas (P6 in Table 6) revealed a high concentration of O, indicating that these dark, dot-like areas were oxides formed by frictional heat accumulation during the wear process. For the C4 coating (Figure 10d), there was an increase in wear debris, and the worn surface showed a mixture of debris accumulation and delamination. Further identification (P7 in Table 6) indicated an increase in the O and Cu content of these wear debris, confirming the presence of accumulated oxidized debris.

During the initial wear stage, the coatings generated a large amount of wear debris, which formed numerous furrows on the worn surface under the action of normal compressive and lateral shear forces from the frictional counterpart. Compared to the C2–C4 coatings containing the Cu element, the C1 coating lacked the toughness and ductility provided by the soft Cu phase [48]. This made the C1 coating more prone to localized stress concentrations under the cutting influence of wear debris, leading to the formation of spalling pits. The EDS analysis results (P1 in Table 6) confirmed that these pits contained mainly Fe, Cr, and Ni elements, indicating that they were caused by the spalling and damage of the coating’s matrix, rather than the oxide film. The toughness and ductility of the coating were improved with increasing Cu content, which reduced the number of pits in the C2–C4 coatings, and this reduction helped to mitigate the volume loss of the coating. In contrast, due to the lower hardness and better ductility of the C2 coating compared to the C1 coating, more furrows (P4 in Figure 10) and adherent wear debris were (P3 in Figure 10) formed on the worn surface rather than obvious flaking pits, resulting in lower wear than that of the C1 coating. As the Cu content increased further, the coatings became more susceptible to oxidation, thus providing more attachment sites for the oxides [20]. Compared to the C1 and C2 coatings, the worn surface of the C3 coating showed fine cutting scratches, microcracks, and embedded wear debris (P5 in Figure 10). These features indicate that the C3 coating with high ductility and elastic recovery ability has better wear resistance, which was consistent with the findings of Yang [20]. However, when the Cu content was too high, the worn surface of the C4 coating became the roughest among the four coatings at RT. Due to its low hardness and load-bearing capacity, the worn surface showed obvious delamination (shown in Figure 10d), making it difficult to form a stable friction layer [49]. This led to severe plastic deformation and abrasive wear mechanisms that increased the wear rate of the C4 coating (shown in Figure 9).

Figure 11 shows the worn morphologies of the coatings at 300 °C. The worn surface features include delamination, oxidized debris, wear debris, spalling pits in the oxide layer, and noticeable cracks. And the delamination phenomenon was more pronounced than at RT. The EDS results in Table 7 indicate that at 300 °C, the O and other metal elements (such as Fe, Ni, and Cu) were higher at different locations in the coatings. This is because the coatings were more susceptible to oxidation during friction at high temperatures.

During the wear experiments at 300 °C, the amount of copper oxide formed in the C1-C3 coatings (P2, P4, and P5 in Table 7) increased gradually with the increasing Cu content. This resulted in denser copper oxide films, which played a positive role in decreasing the wear rate (shown in Figure 9). Moreover, the distribution of furrows on the wear tracks became denser with the increasing Cu content, which was due to the decrease in hardness with the increasing Cu content, leading to more severe plastic deformation [19]. The oxides formed on the surface of the coatings continued to migrate and accumulate under stress cycling at 300 °C. The relatively low content of ductile soft-phase Cu in the C1 and C2 coatings resulted in the limited adhesion of oxide debris, leaving behind substantial wear debris (P2, P3 in Figure 11), oxide islands (P1, P4 in Figure 11), and furrows formed by debris transfer. In contrast, the relative ductile C3 coating retained a more intact oxide film on the surface. This film contained a significant amount of copper oxide (P5 in Figure 11), as well as a small amount of wear debris and pits that were not fully covered by the oxide film. This relatively intact oxide film provided obvious antiwear protection, while the abundance of the copper oxide contributed to the effective lubrication of the friction surfaces [20]. The worn surface of the C4 coating with the lowest hardness was extremely rough with extensive delamination (P6 and P7 in Figure 11d), wear debris, oxide fragments, and large-scale cracks. When the applied frictional force completely exceeded the plastic deformation limit of soft-phase Cu at an elevated temperature, the oxide films formed during the friction underwent severe shear deformation under the influence of frictional forces, which led to extensive cracking and detachment [20]. In summary, at 300 °C, the coatings experienced mainly oxidative wear, and both the plastic deformation and three-body wear caused by the oxides became more pronounced with the increase in Cu content. As a result, the C3 coating had the lowest COF and wear rate (Figure 9).

Figure 12 shows the worn morphologies of the C1–C4 coatings at 500 °C. The oxidation of the coatings was more severe than at 300 °C. The worn surfaces displayed large-scale multilayer structures and fragments, indicating severe plastic deformation during wear at 500 °C. As the thermal softening of the coatings became more drastically at 500 °C than at 300 °C; the oxide layers adhering to the coating surfaces provided less support under the same load, resulting in the severe damage and delamination of the coatings, which exacerbated the volume loss of the softened coatings [50]. The pronounced fluctuations in the COF curves and the increased wear rates observed in Figure 8 and Figure 9, respectively, are also attributed to this more severe oxide layer delamination and fragmentation.

As shown in Figure 12a, the most pronounced delamination occurred in the C1 coating without Ni-coated Cu under severe oxidation conditions at 500 °C. The oxide layer of the C1 coating consisted of iron oxide and nickel oxide. According to the analysis of P1 listed in Table 8, iron oxide dominated the oxide layer, and this oxide film structure tends to be loose and can be easily stripped away by external forces [13,51]. Due to the continuous wear and regeneration caused by the abrasive action on the counterface, severe lamellar delamination and significant COF fluctuations (in Figure 8) were observed in the C1 coating. In the C2 coating where Cu was initially added, the EDS analysis (P2 in Table 8) showed high concentrations of Cu and Ni, with the high-temperature environment of 500 °C promoting the formation of dense oxides of these elements. However, the limited amount additions led to discontinuous oxide films. The dense structure and good ductility of the copper oxide and nickel oxide affected the oxide films, causing its edges to fracture into blocky, fragmented structures under the shearing force of the counterball. In the C3 coating, the higher Cu content enhanced ductility, allowing the wear debris to adhere and form a complete oxide film on the coating surface, reducing the amounts of furrows on the wear scars. However, due to the inherent low hardness of the C3 coating and the effect of thermal softening at a high temperature, significant delamination appeared on the wear scar surface. In addition, due to the complete coverage of the oxide film, loosely expanded oxides similar to iron oxide caused protrusions within the coating, as shown in Figure 12c. For C4 coating, the cyclic impact of the shear forces caused the oxide film to crack, resulting in numerous fractures and the oxide film peeling off into fragments and fine wear debris (Figure 12(d1)). Compared to the Cu and Ni oxide films which were partially retained at 300 °C and provided protection and lubrication (Figure 11d), the probability of failure of the oxide film of the C4 coating at 500 °C was much higher (Figure 12d), leading to a higher wear rate than at 300 °C. In conclusion, at 500 °C, the coatings were dominated by severe oxidative wear, with a gradual increase in adhesive wear with the increasing Cu content. Excessive Cu elements led to oxide film failure, which further increased the wear rate of the coating.

**Figure 13:**
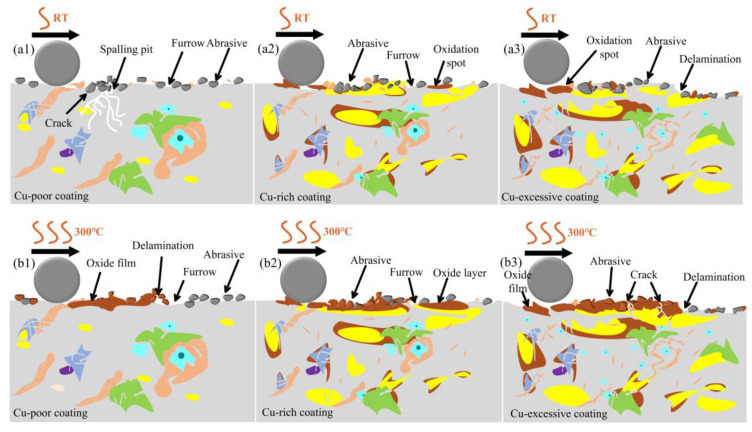

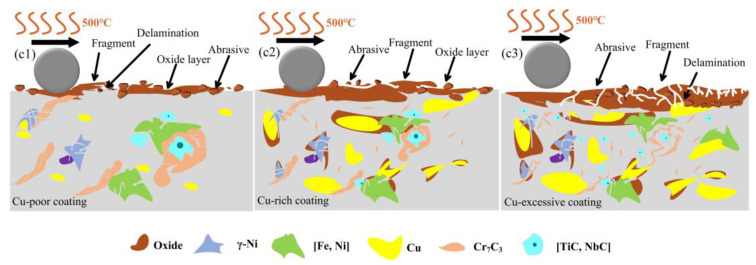
Schematic diagrams of wear mechanisms of the studied coatings. (**a1**–**a3**) Cu-poor, Cu-rich, and Cu-excessive coatings at RT; (**b1**–**b3**) Cu-poor, Cu-rich, and Cu-excessive coatings at 300 °C; **(c1**–**c3**) Cu-poor, Cu-rich and Cu-excessive coatings at 500 °C.

Figure 13 illustrates the schematic wear mechanism diagrams of the studied coatings with different Cu contents at RT, 300 °C, and 500 °C, respectively. At RT, the Cu-poor coating lacked the ductility provided by the soft phase Cu, which led to stress concentration under the cyclic shear of the wear ball, resulting in the formation of various spalling pits on the worn surface (shown in Figure 10(a1)). Additionally, a large amount of wear debris was generated on the coating surface during the RT wear process. The debris was caused by the micro-bulge on the coating surface at the beginning of the wear process as well as by the fracturing and spalling of the coating during the wear process. These wear particles led to the main wear mechanism of all the coatings, which was abrasive wear. In the Cu-rich coatings, the high Cu content provided better ductility and adhesion, and low hardness, which resulted in more wear debris and denser scratches adhering to the coating surface (Figure 13(a2)). As the Cu content increased, the coating was more susceptible to oxidation during wear due to the lower Gibbs free energy of the copper oxide [20]. Consequently, there was a gradual increase in the oxides, with denser dark oxide spots appearing on the worn surface (Figure 13(a1,a2)). However, when the Cu content was excessive, the hardness was too low, which affected the load-bearing capacity of the coating and led to delamination, as shown in Figure 13(a3).

As the wear temperature rose to 300 °C, the degree of oxidation on the coating surface increased. The oxidized wear debris accumulated and was compacted by the wear ball to form oxide films, which had delamination characteristics between the oxide accumulation film and the coating substrate. During continuous wear, the oxide film underwent repeated cycles of adhesion, migration, compaction, and spallation, leading to a large amount of oxide debris adhering to the coating surfaces (Figure 13(b1,b2)). For the coatings with excessive Cu content, the softening at high temperatures caused partial cracking of the oxide film (Figure 13(b3)), as the pressure and shear force exerted by the wear ball on the oxide film surface exceeded its fracture toughness.

The wear at 500 °C resulted in severe oxidative wear on the coatings surface, with large areas covered by oxide layers. However, for Cu-poor coating, the limited copper oxide formation reduced the toughness and lubrication effects, causing severe cracking and multilayer delamination of the oxide layer, with oxide spallation and fragments near the damage area (Figure 13(c1)). With the increase in Cu content, the lubrication and ductility were improved, which reduced the amount of plow furrows and wear debris on the oxide surface (Figure 13(c2)). However, excessive Cu content made the oxide layer more sensitive to temperature and the lack of effective support on the surface of the low-hardness coating could lead to extensive cracks in the oxide layer at higher temperatures due to the pressure from the wear ball, posing a risk of oxide layer failure (Figure 13(c3)).

## 4. Conclusions

Ni60CuMo composite coatings with a Ni60CuMo/IN718 transition layer and a Ni60CuMo/Ni-coated Cu wear-resistant layer were fabricated on the surface of vermicular cast iron RuT400 by laser cladding technology. The effect of Cu content on the cladding quality, phase composition, microstructure, hardness, and tribological properties of Ni60CuMo composite coating were investigated. The main conclusions are as follows:

(1) The coatings maintained good quality without cracks, with phase compositions including γ-Ni, [Fe, Ni] solid solution, dissolved Cu, and carbides such as Cr_7_C_3_, NbC, and TiC. Different Cu content did not significantly impact the phase composition;
(2) Increasing the Cu content led to more pronounced Cu segregation in the solid solution, with morphological changes in the carbides from dendritic to spherical and needle-like structures, indicating a gradual refinement of the microstructure;
(3) The hardness of the wear-resistant layer decreased with the increasing Cu content, while the hardness of transition layer was stable at a high value, resulting in a sandwich structure with a stepped hardness distribution. Increasing Cu content improved ductility and adhesion, reducing wear loss. However, excessive Cu reduced the load-bearing capacity;
(4) The C3 coating exhibited the best wear resistance at RT, 300 °C, and 500 °C. The abrasive wear dominated at RT and the oxidative wear dominated at 300 °C, with the plastic deformation and three-body wear of the coating occurring with the increasing Cu content. At 500 °C, the coatings suffered severe oxidative wear.

## Figures and Tables

**Table 1:** Chemical composition of RuT400 substrate (wt. %).

Element	C	Si	Mn	P	S	Cu	Fe
wt. %	3.60	2.05	0.19	0.011	0.015	0.82	Bal.

**Table 2:** Chemical composition of Ni60CuMo, IN718, and Ni-coated Cu powders (wt. %).

Material	C	Si	B	Fe	Cr	Cu	Mo	Nb	Ti	Ni
Inconel 718	0.05	0.35	0.002	17.50	18.50	0	2.8	5.0	0.66	Bal.
Ni60CuMo	0.5	4.0	4	7.0	17	3	3	0	0	Bal.
Ni coating Cu	0	0	0	0	0	30	0	0	0	Bal.

**Table 3:** The compositions of powder for sandwich-structured composite coatings.

Sample No.	Powder Composition (wt. %)	
	The Transition Layer	The Wear-Resistant Layer
C1	50%Ni60CuMo + 50%IN718	100%Ni60CuMo + 0%Ni-coated Cu
C2	50%Ni60CuMo + 50%IN718	75%Ni60CuMo + 25%Ni-coated Cu
C3	50%Ni60CuMo + 50%IN718	50%Ni60CuMo + 50%Ni-coated Cu
C4	50%Ni60CuMo + 50%IN718	25%Ni60CuMo + 75%Ni-coated Cu

**Table 4:** EDS results of typical structures in different regions in Figure 4 (at. %).

Point	Element Composition									
	B	C	Si	Ti	Cr	Fe	Ni	Cu	Nb	Mo
P1	9.73	20.2	10.22	0.17	2.8	34.47	20.4	1.59	0.09	0.34
P2	8.8	26.75	0.99	0.33	19.83	37.99	3.99	0.12	0.04	1.16
P3	5.68	22.29	0.4	19.45	8.37	5.19	0.19	0.18	31.34	6.92
P4	4.76	14.02	1.33	0.2	25.1	48	5.02	0.15	0.1	1.32
P5	15.03	19.47	5.93	0.25	5.33	28.95	20.8	3.99	0.17	0.08
P6	7.29	23.15	1.48	0.61	29.05	27.27	9.41	0.78	0.09	0.87
P7	3.26	5.86	0.54	1.36	53.65	30.06	3.43	0	0.38	1.47
P8	9.88	25.04	0.85	0.5	26.02	31.1	5.33	0.37	0.09	0.82
P9	9.39	24.35	0.58	0.42	22.65	35.71	5.19	0.13	0.22	1.35
P10	13.02	25.34	5.82	0.24	5.05	23.3	22.49	4.49	0.08	0.16
P11	15.33	22.23	0.98	12.01	13.1	6.1	5.57	0.72	15.26	8.68
P12	18.23	27.92	0.49	0.51	32.77	14.42	4.3	0.11	0.04	1.22
P13	33.03	10.01	2.41	0.24	1.66	13.12	28.49	10.88	0.08	0.08

**Table 5:** The EDS results of the typical structures of the transition layers in Figure 6 (at. %).

Point	Elements Composition									
	B	C	Si	Ti	Cr	Fe	Ni	Cu	Nb	Mo
P1	20.19	19.68	8.03	0.15	3.15	30.22	16.73	1.66	0.08	0.11
P2	9.44	32.48	0.52	0.23	22.93	29.57	2.8	1.15	0.08	0.8
P3	7.17	30.13	0.74	19.32	2.98	1.11	0.53	0.16	33	4.85
P4	8.17	33.39	0.92	0.08	26.53	23.84	6.08	0.12	0.04	0.84
P5	4.14	34.77	0.18	15.46	9.27	1.51	1.19	0.16	26.38	6.94
P6	14.03	28.89	0.3	0.75	20.09	22.98	6.1	1.14	0.04	5.68
P7	39.38	22.62	0.1	0.35	11.04	21.01	4.63	0.4	0.06	0.41
P8	17.12	40.79	1.59	0.12	2.23	10.05	27.84	0	0.13	0.12

**Table 6:** EDS results of each point marked in Figure 10 (at. %).

Point	Elements Composition											
	B	C	N	O	Si	Ti	Cr	Fe	Ni	Cu	Nb	Mo
P1	25.78	0.87	0.87	3.05	1.56	0.05	1.36	17.07	9.02	0.31	0.05	0.05
P2	18.37	2.13	2.13	35.9	0.85	0.17	1.57	8.86	3.42	0.28	0.19	0.14
P3	25.59	24.95	2.67	8.1	1.13	0.06	9.59	17.28	9.33	0.91	0.06	0.33
P4	0	22.15	0	10.89	1.8	6.61	5.47	17.41	20.32	3.54	9.23	2.58
P5	15.13	11.31	2.77	38.11	1.38	0.12	3.3	16.18	10.15	1.35	0.06	0.14
P6	28.42	17	1.47	35.83	0.95	0.13	1.82	9.19	4.57	0.39	0.11	0.11
P7	7.51	6.06	2.8	52.28	1.39	0.18	2.31	11.22	12.58	3.52	0.06	0.09
P8	29.29	20.72	0.23	4.24	1.84	0.13	1.8	15.74	19.99	5.85	0.1	0.07

**Table 7:** EDS results of each point marked in Figure 11 (at. %).

Point	Elements Composition											
	B	C	N	O	Si	Ti	Cr	Fe	Ni	Cu	Nb	Mo
P1	41.87	14.27	2.26	18.38	0.75	2.08	1.95	9.62	5.05	0.5	2.39	0.88
P2	10.87	7.78	3.08	54.23	3.07	0.2	2.12	12.02	5.92	0.53	0.1	0.07
P3	29.32	22.89	1.8	9.82	1.8	0.18	2.54	14.11	14.91	2.34	0.15	0.15
P4	13.42	9.32	3.45	46.59	2.54	0.11	2.15	9.48	11.01	1.8	0.05	0.08
P5	12.73	21.15	2.1	20.55	1.21	0.09	1.57	13.16	11.61	5.64	0.07	0.12
P6	18.06	17.57	2.64	31.16	2.25	0.06	1.78	11.67	12	2.74	0.03	0.05
P7	10.15	6.78	3.72	31.33	2.55	0.41	2.69	16.09	17.69	9.15	0.28	0.17

**Table 8:** EDS results of each point marked in Figure 12 (at. %).

Point	Elements Composition											
	B	C	N	O	Si	Ti	Cr	Fe	Ni	Cu	Nb	Mo
P1	34.65	20.06	2.38	16.25	1.44	0.1	1.88	14.48	7.89	0.64	0.1	0.12
P2	33.1	17.6	1.55	13.89	1.45	0.11	2.61	14.27	13.25	2	0.06	0.11
P3	35.11	20.25	2.6	18.32	1.38	0.09	1.53	11.15	8.25	1.18	0.05	0.09
P4	31.8	14.07	0.83	16.02	1.35	0.18	1.74	11.79	17.27	4.54	0.25	0.15

## Data Availability

Data will be made available on request.
